# The quest for systematization in educational psychology practice—the case of SDQ

**DOI:** 10.3389/fpsyg.2025.1501080

**Published:** 2025-02-14

**Authors:** Thomas Szulevicz, Jon Busck Arnfred

**Affiliations:** Department of Communication and Psychology, Aalborg University, Aalborg, Denmark

**Keywords:** educational psychology practice, Strengths And Difficulties Questionnaire, situated knowledge, systematic knowledge, assessment, normative validity

## Abstract

**Introduction:**

The field of Educational Psychology (EP) practice is currently shaped by debates on the balance between systematized and situated approaches. This study explores these debates through the lens of the Strengths and Difficulties Questionnaire (SDQ).

**Methods:**

Utilizing data from a current research project, the application of the SDQ in EP practice was analyzed. The study involved analyses of SDQ responses and psychoeducational reports and interviews with educational psychologists who used the SDQ in their assessments.

**Results:**

The findings indicate that the participating educational psychologists were generally satisfied with the use of the SDQ. Additionally, a significant portion of the SDQ responses suggested that many of the examined children exhibited symptoms warranting further investigation for ADHD.

**Discussion:**

The article concludes with a discussion on the dual perspectives regarding the systematic use of the SDQ. On one hand, there are arguments for its systematic application On the other hand, while a standardized use of the SDQ ensures systematic information, the questionnaire also contributes to a specific framing of the issue. It is crucial to recognize that the SDQ, originally developed by a child psychiatrist and not specifically designed for EP practice, frames the understanding of issues in a predominantly psychiatric manner. This can lead to a narrow focus on diagnosing and treating perceived deficiencies, potentially overlooking the broader educational and contextual factors that influence student wellbeing.

## 1 Introduction

In recent years, educational psychology (EP) practice has gained significant prominence on the educational policy agenda across many countries (Jimerson et al., 2016; Codding, 2021). This increased focus on EP counseling is driven by several factors, including heightened challenges in educational inclusion, rising expenditures in special education (Thomas and Loxley, 2022), and a growing global consensus about an unprecedented crisis in the wellbeing of children and adolescents (Uhlhaas et al., 2023; Haidt, 2024).

The contemporary emphasis on EP counseling highlights a dual narrative. On one hand, there is optimism regarding the potential contributions of EP counselors in addressing the escalating challenges related to special education expenditures, inclusion, inequity, and student wellbeing. This perspective underscores the preventive and supportive dimensions of counseling, suggesting a positive momentum for EP practice (Splett et al., 2013; Gray et al., 2023).

On the other hand, EP practice faces widespread criticism. Critics argue that EP counseling is overly individual-centric, lacks integration with pedagogical approaches, is deficient in prevention strategies, and suffers from a lack of research and evidence-based practices (Conoley et al., 2020; Kousholt and Morin, 2023; Corcoran, 2024).

This article addresses the latter type of critique concerning the research and evidence base for EP practice. Among others, Lilienfeld et al. (2012), Dombrowski et al. (2022); Dombrowski and McGill (2024) have argued how the EP field is characterized by pseudoscientific practices due to a gap between scientific research and practical application. From this perspective, EPs tend to overrely on personal experience and anecdotical evidence rather than evidence-based practices and systematically collected data.

In contrast, there is also a significant influence within the field of EP from situated and ecological epistemologies (Corcoran, 2024; Røn Larsen, 2024), practice theory (Schatzki, 2017), pragmatism (Burnham, 2013), and Schön’s reflective practice (1987), in which the importance of situated, context-specific expertise and personal judgment in addressing the intricate challenges faced in EP counseling is emphasized.

This highlights two apparently opposing approaches within EP practice: one that pushes for more evidence-based practices, greater uniformity and systematization, and another that values the nuanced, context-dependent insights and reflective practices of practitioners.

The article is based on a study that examines the implementation and use of the Strengths and Difficulties Questionnaire (SDQ) in two EP offices. Psychometric tools like the SDQ are playing a growing role in mental health work and in documenting the effectiveness of services (Frogley et al., 2019). Moreover, SDQ is increasingly used in a Danish EP context as part of an effort to standardize and systematize the data collected about children and adolescents (Szulevicz and Arnfred, 2023b). The SDQ is a brief emotional and behavioral screening questionnaire for children and adolescents, designed to capture perspectives from children, parents, and teachers. It is widely used due to its brevity, ease of administration, and ability to provide an overview of developmental resources as well as emotional and behavioral symptoms.

In this context, the SDQ is used as a prism to explore some of the perspectives from the two aforementioned approaches to EP practice, which, respectively, highlight the need for more systematization versus a more situated approach. Additionally, the SDQ serves as a lens to examine a series of normative, epistemological, and ontological questions currently facing EP practice. While our empirical data demonstrate several favorable opportunities associated with the use of the SDQ, our findings also indicate the necessity for a critical discussion on what its application might represent within the context of EP practice. The article highlights that while a tool such as the SDQ originally designed for purposes like mental health screenings can support systematic workflows in EP practice, it may also lead to a more clinical and/or psychiatric understanding of the challenges faced by children and adolescents.

## 2 Between situated and standardized approaches

Amidst global upheavals, educational psychology (EP) practice confronts mounting pressures, with a surge in students experiencing psychological distress and a corresponding rise in referrals for educational-psychological counseling or assessment (Vivash and Morgan, 2019). There is generally a lack of research that uncovers global patterns and trends in EP practice. However, increasing distress among children and adolescents is widely described as a global trend (WHO, 2021; Höghberg, 2021; Jacobsen, 2024; Haidt, 2024). The causes of this increase are debated and can be found at both individual and structural levels, with societal-level determinants being less empirically investigated compared to individual-level determinants (Jacobsen, 2024).

These trends with increased distress among children and adolescents have revitalized discussions about EP practice, particularly its effects, purpose, resource utilization, and methodological foundations (Müller et al., 2021; Szulevicz et al., 2024), and currently, the need for a systematic foundation of data, research, and information to underpin decision-making processes and evidence-based assessments within EP practice is debated in some of the literature (Dombrowski et al., 2022).

Historically, discussions within EP practice have oscillated between emphasizing context-sensitive approaches and supporting more standardized procedures (Szulevicz and Tanggaard, 2017; Shaw, 2021). The tension lies between a call for situated expertise, where procedures are tailored to specific contexts, and a demand for systematic, evidence-based practices. This dichotomy reflects ongoing debates about the optimal balance between professional autonomy and methodological rigor within EP practice.

Proponents of situated approaches emphasize the importance of allowing practitioners to tailor their methods to the specific needs and circumstances of each case. From this perspective, a flexible and context-sensitive approach is crucial as it respects the local dynamics and complexities inherent in each situation. Drawing on ecological epistemologies (Corcoran, 2024), and theoretical frameworks like practice theory (Schatzki, 2017), pragmatism (Burnham, 2013), and Schön’s concept of reflective practice (1987), advocates highlight the importance of context-specific expertise in navigating the complex challenges encountered in EP counseling. A situated perspective argues that meaningful practice is contingent upon practitioners’ ability to adapt to the distinctive nuances of each case, rather than rigid adherence to abstract principles. The situated approach has also been brought into focus by the fact that many EPs are spending increasing amounts of time writing psychoeducational reports of children’s special educational support needs. Much of this work involves documentation, where the EP is distanced from the practices and everyday lives in which the children are engaged, and, on that basis, there has been a greater interest in making EP practice more situated and practice-oriented (Burns et al., 2015; Kranzler et al., 2020; Szulevicz and Arnfred, 2023a).

The situated approach is in many ways also skeptical of what is often described as more mainstream approaches to EP practice, highlighting that these ways of working often perpetuate systemic inequalities by adhering to standards that fail to account for diverse student experiences and contexts. The reliance on such standards might even restrict the opportunities available for adapting procedures and interventions to the concrete situations. The reliance on such standards might as well limit the flexibility needed to tailor procedures and interventions to the specific circumstances of each situation (Begon and Billington, 2019; Corcoran, 2024; Røn Larsen, 2024).

However, critics of a situated approach argue that reliance on subjective proficiency alone without utilizing evidence-based research that has emerged from well-conducted studies poses significant risks for bias in approaches to and assessments of children in difficulties. Although the value of professional judgment is acknowledged, it is emphasized that such judgments should be refined by routine access to information about the outcome in each case (Kahneman and Klein, 2009). It is further highlighted that school psychology generally lacks such systematic feedback loops (Dombrowski et al., 2022). According to Dombrowski et al. (2022), many school/educational psychology practitioners still engage in so-called low-value assessment practices, defined as those that: “(a) have limited evidence for their clinical utility, (b) are not the most effective available practice, (c) have an unacceptable risk of adverse effects, or (d) are diagnostically or therapeutically unnecessary.” Consequently, educational psychology practice has been scrutinized for perceived pseudoscientific tendencies, characterized by anecdotal or subjective evidence and interventions lacking empirical validation. Issues such as confirmatory and conjunction bias are highlighted as problematic (Lilienfeld et al., 2012; Dombrowski et al., 2022; Braden, 2024).

In response to these criticisms, there is a growing momentum toward evidence-based practice (EBP) within educational psychology (Kratochwill and Shernoff, 2004; Robinson et al., 2018; Dombrowski et al., 2022). EBP advocates for the integration of research evidence, professional expertise, and contextual considerations in decision-making processes. This approach emphasizes rigorous evaluation of interventions through empirical research methods, fostering transparency, accountability, and ethical responsibility within the field.

In recent decades, there has been significant advocacy worldwide for EBP, endorsed not only at the political level but also by numerous professional associations. For instance, the American Psychological Association (APA) has championed the call for a more robust evidence foundation in school psychology practices (Kratochwill, 2007).

However, the adoption of evidence-based approaches is not universal across all contexts. In regions like the UK, Ireland, and Scandinavia, there is relatively less emphasis on EBP in education, with EPs often prioritizing utility and social value over established evidence bases (Burnham, 2013; Szulevicz and Tanggaard, 2017). Nevertheless, there is an increasing orientation toward implementing more evidence-based and systematic methods in EP practice within the Scandinavian context.

## 3 The Strengths and Difficulties Questionnaire (SDQ)

We now aim to focus more specifically on the SDQ questionnaire. The rationale behind this lies in the already mentioned trend that psychometric measures generally play an increasingly important role in mental health work and in documenting effectiveness of services (Frogley et al., 2019). In a Danish context, the SDQ is also increasingly being promoted as a tool for ensuring systematic information gathering in EP practice (Szulevicz and Arnfred, 2023b). In the context of this article, SDQ is of interest precisely because its utilization can be seen to align with some of the forces advocating for the need for a more systematic EP practice. Evidently, this does not mean that the use of the SDQ in EP practice captures all the nuances in the discussions regarding the need for more situated or standardized approaches in EP. Rather, a closer analysis of the application of the SDQ can be used for an empirical analysis of some of the potentials and limitations associated with the systematic use of such a tool.

The SDQ is an abbreviation for the “Strengths and Difficulties Questionnaire.” The SDQ is a short questionnaire that focuses on the mental wellbeing and functioning of children and adolescents. It was developed shortly before the turn of the millennium for use in surveys (Goodman and Scott, 1999). Since then, the tool has gained global popularity and is now available in authorized translations in more than 80 languages. The questionnaire can be answered by parents or other caregivers as well as by educators and teachers. There is also a self-report version aimed at 11–17-year-olds.

As the name suggests, the 25 questions that are always answered address both resources and potential signs of difficulties. These questions are followed by a question about whether there are perceived difficulties overall in one or more of the following areas: emotions, concentration, behavior, or interaction with others. Here, the response can be graded on a four-point scale from “No” to “Yes, severe difficulties.” If the respondent believes there are some difficulties, a few additional questions are asked about duration and how much the difficulties affect wellbeing and functioning in daily life. In the Danish electronic version, it is indicated how the scores compare to a large and representative Danish norm material covering children in both mainstream and special education settings (Arnfred et al., 2019). However, scores only provide a very general and approximate summary of the collected responses. Like all other questionnaires, unless a calculated score is either zero or the maximum possible, the number contains no information about how the individual questions were answered.

The characteristics described above suggest that the SDQ can be a useful tool in EP practice for broad and systematic gathering of preliminary information from key persons about the resources the child shows in everyday life and how he or she copes with ordinary challenges. However, its applicability in EP practice also deserves critical scrutiny Although it may initially sound convincing that a questionnaire like the SDQ offers a greater degree of systematization in EP practice, it is also important to be aware that the tool was developed in a child and adolescent psychiatric context, and it is therefore not a questionnaire specifically designed for EP practice. Using a questionnaire developed for a different professional framework by a child psychiatrist might inadvertently shift the understanding of children’s difficulties from primarily pedagogical frameworks to more psychiatric ones. This shift risks promoting a one-sided, diagnostic, and individual focus on students, potentially overlooking specific challenging factors in children’s educational everyday lives.

Moreover, a systematic use of the SDQ in educational psychology must be approached with caution. Like other questionnaires in EP practice, there is a need for substantial professional attention to interpreting its results, with a clear understanding that the SDQ does not necessarily address all relevant circumstances. The risk is that by focusing on specific aspects, the questionnaire might overlook significant factors unless these are addressed through other means. Building on the previously mentioned point that individual-level determinants are more frequently examined in empirical studies than societal-level determinants (Jacobsen, 2024), a similar risk may be associated with the use of the SDQ. The questions in the SDQ primarily focus on individual determinants, potentially overlooking contextual, structural, educational, and societal factors that influence the child’s situation.

Furthermore, research has also documented that the SDQ cannot stand alone as a tool to measuring the mental health and emotional wellbeing of young people (Wright et al., 2019).

The use of any instrument or technology like SDQ thus requires careful consideration of what the tool highlights and what it may inadvertently ignore, which specifically relates to this article’s focus on the relationship between systematic and situated approaches to EP practice. If the SDQ is applied stringently and authoritatively, with an overly narrow focus on scores, there is a risk of overlooking contextual or other explanatory factors that might be modified by intervention.

Given these reservations, the SDQ’s characteristics as an accessible, broadly covering, and well-accepted questionnaire are probably significant reasons why the questionnaire is currently gaining increasing acceptance in EP practice contexts.

## 4 The use of SDQ in educational psychology practice

There is plenty of research that has utilized the SDQ to investigate and document development in larger populations as psychometric properties based on calculated scores have been documented in several reviews (Emerson, 2005; Vostanis, 2006; Warnick et al., 2008; Stone et al., 2010; Kersten et al., 2016; Bergström and Baviskar, 2020). The latter of these reviews do, however, draw attention to differences when the questionnaire is filled out by mothers and fathers, respectively (Bergström and Baviskar, 2020). There is also some research that has examined the clinical relevance of the SDQ in various healthcare contexts (Mølland et al., 2023).

However, there is not much research that has uncovered how and to what extent EPs use the SDQ in their daily practice as a clinical and educational tool. Nevertheless, Jenkins et al. (2014) describe from an American perspective how there is generally increased focus on schools being responsible for students’ social and emotional development, and that this work is carried out in close collaboration with EPs. In this context, universal screening tools are increasingly used, which can quickly, effectively, systematically, and on a psychometrically sound basis, identify students who may need support or further assessment (Moore et al., 2023). Jenkins et al. specifically mention five different short screening instruments for assessing mental health among students, of which the SDQ is one. Jenkins et al. (2014) highlight that the SDQ is very accessible, manageable, inexpensive, and easy to score and calculate. However, it is also emphasized that a published manual is missing, and that the easy accessibility of the questionnaire carries the risk that parents or other laypersons may try to interpret the questionnaire’s results without consulting a mental health professional.

Zee and Rudasill (2021) also describe that there is great potential for EPs in the use of SDQ in relation to students exhibiting internalizing symptoms and students who present problematic behaviors.

From a British context, Lowther (2013) argues that there is generally a lack of tools that can help EPs systematically assess and evaluate the effects of their interventions. In this context, it is highlighted how the SDQ could potentially be a tool for this purpose. Based on interviews with EPs, several psychologists expressed some initial skepticism about the use of the SDQ, with concerns among psychologists that the questionnaire was too broad and would not be able to capture the complexity and nuances of the processes. However, the interviews also indicate that the questionnaire was considered relevant because it gathers information from multiple informants about the child.

In a Danish EP context, EPs are increasingly offering therapeutic treatment to children and adolescents in psychological distress. In this context, there is a declared political intention from the Ministry of Children and Education that such interventions should be preceded by a thorough evaluation, using the SDQ or similar validated tools:

“It is essential that educational psychologists use standardized and knowledge-based tools and structure the process for the professional assessment in a way that ensures that the assessment can be scaled depending on whether there are more or less complex issues and consequently varying degrees of severity, so that the possibility of interdisciplinary involvement is ensured if needed. For example, it may be relevant to begin by using the Strengths and Difficulties Questionnaire (SDQ) and supplement with other validated assessment tools, followed by a conversation/interview with the child/the youth and the parents and then a case formulation with a holistic analysis of the current problem, including the specific context.” (Danish Ministry of Children and Education, 2020).

From an educational policy standpoint in Denmark, there is thus an articulated expectation that EPs use the SDQ or other similar questionnaires to systematically measure the effectiveness of interventions. And even though not all EP offices systematically use the SDQ or similar questionnaires, there is thus an increasing expectation for EPs to employ more systematic methods of information gathering. And despite the fact that EPs have, of course, always used various tests, questionnaires, and other methods to collect information more or less systematically, a political focus on the appropriateness and quality of the methods used stands out as something new. This trend underpins the relevance of the current study, which aims to qualitatively investigate how two different EP offices utilize the SDQ and how the EPs at these offices perceive the use of the questionnaire.

## 5 Method

This research project is a 2-year exploratory and empirical investigation focused on the implementation and utilization of the Strengths and Difficulties Questionnaire (SDQ) in two Educational Psychology (EP) offices. This research was approved by Aalborg University’s contracting unit. Informed consent was obtained from all individual participants included in the study.

In one participating municipality, all municipal EPs were involved in the project, while in the other municipality, only a subset of EPs participated. To gain comprehensive insights into the work practices and the use of SDQ within the participating EP offices, we collected a total of 117 anonymized psychoeducational reports from one office and 17 anonymized reports from the other.

There is a clear methodological shortcoming in the design due to the significant differences between the two participating municipalities in several parameters, such as the number of psychoeducational reports, the availability of SDQ responses, and the number of interviews. However, the aim of this article has never been to conduct a rigorous comparative analysis of the two municipalities. Instead, it seeks to view them as distinct contexts for EP practice.

Some of the psychoeducational reports from the two municipalities included SDQ data, while others did not. Additionally, we obtained electronic access to all SDQ questionnaire responses handled by the EP offices.

As part of the research project, support for integrating the Strengths and Difficulties Questionnaire (SDQ) into EP practice was offered. This support included two teaching sessions that focused on the characteristics and application of the SDQ. The teaching was primarily conducted by the second author of the article, who has extensive experience with the SDQ questionnaire. It began with a brief theoretical introduction to the SDQ, followed by a focus on the practical application of the questionnaire. During the sessions, EPs were encouraged to use the questionnaire as early as possible in their EP practice and to gather responses from as many respondents in both school and home settings as possible. The teaching was kept as neutral as possible but highlighted the potential benefits of a systematic use of the SDQ. It cautioned against a reliance only on scores and encouraged the EPs to look at how individual questions were answered by each respondent to be used as a basis for further information gathering in order to fully understand the resources and possible challenges observed in different contexts.

Additionally, we conducted two practice-oriented workshops at each office. During these workshops, discussions with EPs centered on their experiences and challenges with the SDQ, drawing on the project’s initial observations and results, which were reviewed collectively. EPs also had access to ongoing support aimed at optimizing the utilization of collected SDQ responses.

To delve deeper into EPs’ experiences with SDQ, we conducted 13 qualitative interviews. The interviews were conducted using an interview guide that specifically focused on the psychologists’ experiences with the SDQ. The guide also explored some of the patterns identified in the collected SDQ responses. For the interviews, all eight educational psychologists from municipality 1 participated, while five educational psychologists with experience working with the SDQ participated from municipality 2.

Table 1 provides a schematic representation of the research project’s overall empirical data foundation.

**Table 1:** Empirical data foundation of the project.

Data source/activity	Psychoeducational reports	SDQ responses	Workshops/teaching sessions	Interviews
Data from Municipality 1	24	1,114	4	8
Data from Municipality 2	93	205	4	5

Braun and Clarke’s (2012) thematic analysis approach was employed to analyze the interviews.

The thematic analysis was conducted in three stages:

Data collection and transcription: The first author conducted the interviews, which were then transcribed verbatim.

Familiarization with data: The second stage involved reading through the transcribed interviews to identify initial codes and patterns. Using Braun and Clarke’s (2012, 2024) approach, we generated initial codes by systematically highlighting significant features across the dataset. Codes were labeled based on their relevance to the use of SDQ and the frequency of occurrence. For example, most EPs highlighted the questionnaire’s manageability and its ability to provide a quick and relevant overview of several key aspects related to the child. Additionally, one of the most frequently mentioned reservations was whether the questionnaire might contribute to an overly psychiatric understanding of the child’s challenges. After initial coding, we reviewed and refined the codes, merging similar ones (like manageability and understanding of challenges) and discarding less pertinent ones. For example, throughout the interviews, some EPs individually mentioned aspects related to the questionnaire’s connection with other questionnaires or the managerial support for the use of the SDQ. While these points could certainly be relevant, they were excluded if they did not recur across multiple interviews.

This process helped in clustering related codes together, which facilitated the identification of broader themes.

Identification of cross-cutting themes: All transcribed interviews were compared to identify overarching themes and patterns. The primary focus in this stage was to understand how the EPs assessed the use of the SDQ.

Inspired by Braun and Clarke (2024), we aimed to maintain a clear and consistent methodology. We worked inductively to identify themes based on the coding process, and by ensuring that our theoretical assumptions were explicitly stated and transparently engaged. This approach was evident early in the analysis when we introduced the relationship between standardized and situated approaches to EP practice as an analytical tool for working with our empirical material. We also reflected on our own preconceptions and how they might influence the analysis, striving for critical self-awareness. Thus, we attempted to base our work on our different starting points. The first author of the article is an educational psychologist and had no prior knowledge of the SDQ before the research project, while the second author has a professional background as a medical doctor and has extensive clinical and research knowledge of the SDQ.

## 6 Results

The subsequent sections present several significant findings derived from (1) the compiled SDQ responses in the research project and (2) the qualitative interviews conducted with EPs and the psychoeducational reports.

### 6.1 SDQ responses

Table 2 provides an overview of the SDQ (Strengths and Difficulties Questionnaire) responses from Municipality 1 and Municipality 2, including the number of responses, follow-ups, responses from parents and teachers/caregivers, self-responses from children over 11 years old, and the average number of responses per case.

**Table 2:** SDQ responses.

Aspect	Municipality 1	Municipality 2
Total Number of SDQ Responses	1,123 (pertaining to 290 children aged 5–17 years)	207 (pertaining to 85 children aged 6–17 years)
Number of Cases with Follow-ups	37 (averaging 8.5 months)	None
Parent Version Responses	480 (average of 1.5 responses)	98 (average of 1.1 responses)
Teacher/Caregiver Version Responses	515 (average of 1.6 responses)	90 (average of 1.1 responses)
Self-Responses for Children over 11 years old	128	19
Average Number of Responses	3.4 (including self-responses)	2.4 (including self-responses)

The significant difference in the number of SDQ responses between the two municipalities is attributable to differing implementation practices. In municipality 1, all EPs were required to use the SDQ as a standard tool for every child registration. This mandatory use was the result of a management decision to integrate the SDQ into all case processes within the EP office. Conversely, in municipality 2, the use of the SDQ questionnaire was voluntary. Consequently, only those EPs who opted to use the questionnaire had their SDQ responses registered.

Below, we present several results from municipality 1, as the mandatory collection of SDQ responses from this municipality provides a good basis for systematic analyses.

The 1,123 SDQ responses concerning 290 children (of whom 70% were boys) were collected from March 2019 to March 2022. The average age at the time of the first set of SDQ responses was 10.8 years.

As outlined above, an average of 3.4 respondents provided SDQ responses when such responses were asked for initially, or as part of a follow-up. Almost half of the times (148), separate responses were obtained from both the mother and the father. Additionally, in 156 cases, responses were received from more than one teacher or educator. In approximately half of the cases (144), the child was over 11 years old at the time of the initial SDQ responses, and in 128 of these cases, self-reports were included. Overall, the data indicate that the SDQ questionnaires effectively collected responses—often multiple—from both school and home environments, as well as from the children themselves when they were over 11 years old.

In almost 90% (245) of the initial SDQ submissions, at least one response indicated either an elevated or highly elevated impact score, i.e., a score seen in less than 10 or 5% of responses in the normative sample. In 89 cases (31%) impact scores from both parents and teachers/educators, along with the sub-scores for hyperactivity and attention difficulties pointed to a statistical likelihood of more than 50% that the criteria for ADHD would be met if systematically assessed. In a further 108 cases (38%), the combination of scores pointed to a lesser but still significantly elevated probability for difficulties similar to ADHD. These estimates are based on algorithms originally developed by Goodman et al. (2000a) and Goodman et al. (2000b).

The utility of these algorithms has since been verified in a number of contexts (Mathai et al., 2004; Brøndbo et al., 2011; Russell et al., 2013; Rimvall et al., 2014; Posserud et al., 2014). In contrast to the high prevalence of ‘likely’ or ‘possible’ ADHD in the consecutive EP cases, similar combinations of scores were found in 1.3% and 7.0%, respectively, in the large Danish normative material mentioned earlier (Arnfred et al., 2019).

To our knowledge, the prevalence of specific developmental delays matching the criteria for ADHD among children referred to EPs has not previously been systematically estimated. Since abnormally rapid exhaustion of attention and impulse control cannot be reliably recognized by testing or professional observation, the diagnosis relies on detailed information from observations made by parents and teachers over an extended period. To determine if such problems might be present, it is necessary to ask parents and teachers how the child typically copes with specific challenges. A conservative interpretation of the underlying SDQ scores and their applicability to the collected data from Municipality 1, where SDQ-responses were routinely collected results in an estimate that in between one quarter and one third of the consecutive cases involved children facing difficulties that match the criteria for ADHD.

In the electronic platform used for collecting and presenting SDQ responses the EPs could opt to see automatically generated comments that might include information about how a combination of scores in the collected SDQ responses were statistically associated with persistent self-regulation challenges. These comments emphasized that no conclusions can be drawn from the answers alone in individual cases, but that the said combinations might indicate the relevance of more in-depth information gathering to shed light on the child’s self-regulation abilities.

### 6.2 Qualitative interviews and analyses of psychoeducational reports

We conducted 13 semi-structured interviews with EPs from the two collaborating offices, focusing on their experiences with and utilization of the SDQ questionnaire. Eight of the interviewed psychologists hailed from municipality 1, where extensive familiarity with the SDQ exists, while the remaining participants from the other municipality had used the questionnaire less consistently.

For space reasons, we are unable to provide detailed examples from the results of the analyses. Therefore, we have summarized the main themes derived from the interviews with the EPs in Tables 3, 4. Overall, we have distinguished between positive experiences with the questionnaire and comments pointing to limitations and possible biases.

**Table 3:** Themes from interviews (potentials).

Theme	Potential
Initial tool	Provides relevant and quick overview Good for giving an initial overview Matches well with information from network meeting
Manageability/usability	Easy to administer in the digital version A manageable questionnaire An easily administrable form Clear because responses can be compared Easy to gather responses from respondents Can be used as a tool for reassessments in the special education field
Information gathering/data collection	Provides a good overview of a situation Valuable for uncovering different assessments from professionals and parents Good follow-up tool The automatic comments are helpful Counteracts bias in understanding and decision
Dialogue tool	Provides a basis for dialogue with students during completion Can promote a child perspective (in connection with self-reports)

**Table 4:** Themes from interviews: (reservations).

Theme	Reservations
Understanding of challenges	Can contribute to a focus on child psychiatric issues Auto-generated comments do not consider factors such as upbringing conditions or other contextual circumstances Auto-generated comments often point to ADHD May be too sensitive regarding behavioral difficulties, as it takes very little in teacher responses for a score to be slightly elevated Might lead to a quantified/‘scorefied’ notion of children’s challenges
Manageability/applicability	Rarely contributes new information about the child/student—and can therefore seem superfluous or redundant to use. There can be a risk that collaborating partners are using scores from SDQ inappropriately
SDQ as a follow-up tool	The SDQ has limited sensitivity to function as an evaluation/follow-up tool Follow-ups in EP practice are a good idea, but a questionnaire like SDQ might not be the most appropriate tool.

As representative examples of some of the positive experiences, the psychologists expressed the following:

- I think it is a good tool. I believe it provides a good, quick, and easy overview of whether there are any reasons for concern.
- I like it. I think it makes sense, it provides an overview, it’s easy to send out, and I believe you get information quite quickly on some very important areas. I particularly like that it helps to understand to what degree problems affects the children; how much it interferes with their daily lives, their learning, and their social interactions.

In Table 3, we have summarized some of the main trends from the interviews, where EPs express positive experiences with the SDQ.

Overall, the positive evaluations of SDQ were prevalent among EPs in the 13 interviews with positive comments outweighing more skeptical ones. The EPs were satisfied with the relatively simple and condensed format of the questionnaire, and they also reported that it was easy to administer. Despite the relatively few questions, they mentioned that it still managed to inquire about many relevant dimensions. Finally, the EPs were pleased with the questionnaire’s ability to gather information from both school and home, as well as from children and adolescents over 11 years old.

Concerns about the use of SDQ in EP practice were also mentioned during the interviews. The EPs particularly noted that the combined responses often resulted in scores for attention problems and impact scores indicating that criteria for ADHD would be met. Several EPs expressed concern about a potentially dominant psychiatric framework for understanding children’s and adolescents’ difficulties.

Somewhat surprisingly our analyses of the psychoeducational reports using the SDQ revealed a persistent tendency toward primarily using the calculated numbers and their relation to the norms. It had repeatedly been emphasized in connection to the above-mentioned teaching and workshops that to fully realize the potential of the SDQ, the scores should only be used as a help to identify key questions and focus on how different respondents answered them. The observed practice, however, seemed to reflect a deeply ingrained approach to the use of questionnaire results. Consequently, scores were generally given significant importance, while the SDQ’s potential as a tool for fostering dialogue based on the respondents’ observations in various situations was very sparingly explored.

Below are a couple of interview examples where some of the interviewed EPs critically reflect on a potential systematic use of the SDQ in EP practice and whether the questionnaire promotes a psychiatric understanding of children’s challenges.

- It may also be that sometimes you will discover things you had not considered… But… but I actually think it’s something… I would have asked about otherwise. Like attention and interaction with peers and… yes. I actually think the SDQ can be meaningful in EP practice. But I am doubtful whether it is meaningful to use it systematically in all cases.
- But I also think that the SDQ to some extent actually contributes to creating this focus. That there is a focus on some of the child psychiatric issues. If the SDQ had stopped after the impact and wellbeing and emotional symptoms, and then had something simple: How is learning going, how is… does the child have a friend, and so on—then I do not think it would create the same psychiatric focus as when specifically asking about hyperactivity and social attention level.

Table 4 summarizes some of the primary concerns expressed by the EPs during the interviews.

## 7 Discussion

The application of the SDQ can be both illustrative and limited in terms of shedding light on issues related to the knowledge base, information collection methods, and systematic approaches in EP practice. On one hand, the use of the SDQ in two Danish municipalities provides only a very localized and specific picture of practice within these two authorities. Additionally, the SDQ represents only a limited portion of the questionnaires, tests, and other information-gathering methods available to EPs. The present study is thus narrow in scope, and the use of the SDQ only provides a limited insight into what a systematic use of questionnaires means for EP practice.

On the other hand, the use of the SDQ is illustrative in highlighting some of the advantages and disadvantages that standardized use of specific, predefined questionnaires can offer. Most EPs generally expressed satisfaction with the SDQ as a questionnaire in relation to EP practice. Therefore, based on the study, it can be suggested that most EPs consider the SDQ a relevant questionnaire for use in EP practice.

However, the study also prompted some critical perspectives on the use of the SDQ, which we will discuss in the remainder of the article.

Firstly, it is essential to recognize that no questionnaire produces neutral knowledge. Like all other methods of documenting and mapping social practices, questionnaires are influenced by a specific way of delineating the phenomenon under investigation. This is what Biesta (2015) has termed the normative validity of information gathering in another context. Secondly, we will discuss how the data produced by the SDQ is utilized. In this context, we will address the significant tendency to use the questionnaire scores in a score-based manner. Finally, we will discuss what the study can contribute to broader discussions about EPs’ methodological grounding.

### 7.1 The normative vs. the technical validity of SDQ

As mentioned previously, psychometric measures are playing a progressively prominent role in mental health work and in documenting the effectiveness of services (Frogley et al., 2019). Given the growing use of the SDQ questionnaire in Danish EP practice, the purpose of this study was to empirically investigate the impact of SDQ utilization on EP practice.

We have argued that the field of EP is characterized by two different approaches. The first approach advocates for a greater application of systematic methods in the EP field. The second approach emphasizes a situated perspective, arguing that standard methods should not be identified *a priori*. Instead, the EP should determine the methodological basis of their work and interventions in a more situated and context-based manner.

To help discuss some of our findings, we now turn to the Dutch educational philosopher Gert Biesta (2015), who, in a different context, writes about the PISA surveys, stating that they possess both technical and normative validity. Technical validity refers to whether the survey provides an accurate picture of what it actually measures. For example, in the context of the PISA surveys, technical validity would assess whether the tests employed accurately measures students’ reading, mathematics, and science skills. Conversely, the survey’s normative validity refers to whether the survey represents an appropriate and acceptable operationalization of the quality of the school system. This involves questioning whether the PISA tests measure what we consider important in terms of educational values and goals. For instance, do the PISA tests reflect the broader educational aims usually associated with good education, such as critical thinking and creativity (Biesta, 2015).

Similarly, there can be said to be a corresponding validity discussion regarding the use of SDQ in EP practice. SDQ’s technical validity is well-regarded, as the questionnaire is one of the most validated and widely used globally. In many ways, a normative validity discussion of SDQ’s potential and justification in EP practice is more interesting, as it raises questions about the fundamental purpose associated with the use of SDQ in an EP context. For example, what are the consequences of a systematic use of the SDQ in EP practice for the understanding of children’s and adolescents’ challenges? Returning to our results, in 31% of our cases, both parents and teachers/educators reported impact scores and sub-scores for hyperactivity and attention difficulties that suggested a greater than 50% chance of meeting the criteria for ADHD upon systematic assessment. Additionally, in 108 cases (38%), the scores indicated a moderately high probability of exhibiting difficulties corresponding to ADHD. In this study, the systematic use of the SDQ revealed that a significant proportion of the assessed children exhibited symptoms of attention difficulties warranting further investigation. In most cases, this would involve considerations regarding referral for psychiatric evaluation. On one hand, it can be argued that the systematic use of a questionnaire like the SDQ is beneficial, as it might lead to fewer children with ADHD symptoms being overlooked.

However, it can also be contended that the systematic use of the SDQ may influence and shape EPs understanding and perceptions of children’s challenges in a more psychiatric direction. This concern was highlighted by several respondents in the qualitative interviews. Such a trend is noteworthy as it raises more fundamental discussions about the purpose and function of EP practice and the role of EPs. The general increase in the number of children and adolescents diagnosed with ADHD is highly debated, and a concern for over-diagnosis of ADHD is raised (Tam et al., 2024). With the incidence of mental health disorders, including ADHD, in increase (Whitney and Peterson, 2019), it is an interesting question how EPs should approach these trends. Our empirical data indicates that the systematic use of the SDQ may potentially make EPs more inclined to include a psychiatric framework in their understanding of children’s and adolescents’ challenges.

Returning to Biesta, he argues that the ways assessment tools are used are not neutral. Instead, they are embedded with specific normative assumptions about what constitutes valuable knowledge. For the PISA tests, it means that the way the PISA surveys are organized and interpreted reflect and reinforce particular educational and societal values. And our point is that the same type of attention should also apply when using assessment and information gathering tools (like for example the SDQ) in EP practice.

Focusing on the normative validity of the SDQ is particularly important in this context. It raises the question of whether the SDQ might contribute to reinforcing specific interpretations of children’s challenges.

This also relates to the question of the requirements for the use of specific and more systematic tools and procedures in EP practice.

From proponents of the need for more systematic practices within EP, it is argued how it is generally more beneficial to use standardized and validated instruments than to rely solely on the personal preferences of individual educational psychologists. Nevertheless, it is worthwhile to revisit Biesta’s insights. In his critique of standardized assessments, he asks: What is the purpose of education, and are we measuring what we truly value, or do we risk valuing only what can be measured?

In a similar vein, it might be considered: What is the ultimate goal of EP practice? Is it to accurately understand each child’s challenges within a framework aligned with psychiatric and diagnostic methodologies? Or should the practice of educational psychology be more pedagogical and didactic, focusing on understanding a child’s potential for participation in an educational setting? While there is no need for a strict dichotomy between psychiatric and pedagogical approaches, it is essential to recognize that if the SDQ is widely adopted as a standardized tool within EP practice, it is a tool that originates from a child psychiatric framework. This does not, of course, render the tool irrelevant in EP practice. Rather, it highlights that the requirements for systematic approaches and the use of specific professional tools like the SDQ also serve to frame the understanding of issues in particular ways, which is essential to be aware of.

### 7.2 Quantified use of SDQ scores

As already mentioned, we gained access to a number of psychoeducational reports in which the EPs reported their use of the SDQ questionnaires. In this context, it became clear that the SDQ questionnaire was primarily used to report scores in various areas. These scores were rarely placed in a broader context, nor were discrepancies in responses from teachers and parents followed up on.

Overall, the presentation of the responses to the SDQ questionnaire was thus merely ‘scorified,’ or quantified and these scores were neither contextualized nor explained in any other way.

This can be explained by the heavy workload on EPs, which often necessitates streamlining the time spent on report writing (Szulevicz and Arnfred, 2023b). Conversely, it may also be explained by professional habits, where numerical scores and data are given more value and legitimacy than qualitative descriptions. From a sociological perspective, this can be said to be part of a larger trend in which an increasing number of phenomena, particularly social phenomena, are translated into numbers and used to evaluate and rank all sorts of social practices and behavior (Mau, 2019). For the EPs, the numbers are probably considered more authoritative if a child is referred to a child and adolescent psychiatric assessment. The problem however is that such a reductionist use of SDQ might lead to a reduction of complex psychological, behavioral and educational processes to simplistic metrics, and it shifts focus from understanding the individual answers to the scores. Such a practice would likely also be considered a low-value assessment practice (Dombrowski et al., 2022), and this trend toward quantification can lead to an over-reliance on numerical data, potentially misrepresenting the nuanced realities of individual cases.

This critique is not directed specifically at the SDQ questionnaire itself, but rather at a particular way it is utilized. The SDQ is a screening tool that provides an overview and can highlight areas warranting further investigation. However, it is not designed to have its scores used authoritatively. The fundamental principle that professionals should be aware of the limitations of their assessment procedures and the information obtained from them is central to ethical assessment practice (Reynolds et al., 2021). This consideration is also relevant in discussions about evidence-based demands for more systematic and standardized methodological rigor in educational psychology practice. While systematic approaches can be a worthy goal, it does not inherently guarantee professional quality. It is important to consider how assessment methods frame the understanding of issues, as well as to be mindful of how assessment results are used, interpreted, and communicated. The results of this study show that a systematic use of the SDQ contributes to uniformity in data collection, but this systematic approach also imposes a specific normative framework for understanding children’s challenges.

## 8 Conclusion

In this article, we have examined how debates between more systematized and more situated approaches influence the field of EP practice. Based on a current research project, we have used the application of the SDQ questionnaire in EP practice as a prism to analyze questions and issues related to a more systematic use of such a tool. Our data showed that the participating EPs were generally pleased with using the SDQ. Another significant finding was that a large portion of the SDQ questionnaires indicated that the examined children have challenges suggesting that further investigation for ADHD symptoms might be considered.

The article concludes with a discussion where, on one hand, it is argued that there can be reasons to consider a more systematic use of the SDQ. On the other hand, we also point out that a questionnaire like the SDQ, which was not developed specifically for EP practice, may contribute to framing the understanding of issues in psychiatric terms, which might not be suitable in an EP context. This, of course, requires significant professional attention.

A notable limitation of our study is that it is based solely on two Danish municipalities, and our analyses of the use of the SDQ only address one aspect of EP practice. Therefore, the intention of this article has not been to draw definitive conclusions about the use of the SDQ in EP practice or the advantages and disadvantages of EP approaches that primarily emphasize systematic or situated approaches. Nevertheless, our empirical analyses provide an interesting testimony that the systematic use of the SDQ certainly provides relevant information about a child, but it also may point to a possible psychiatric understanding of the child’s situation. Historically, the EP field has frequently engaged in discussions regarding its raison d’être, with debates focusing on whether it should move in a primarily clinical or educational direction (see, for example, Reger, 1964). If questionnaires like the SDQ are not used with sufficient critical attention, there is a significant likelihood that the systematic use of such tools will steer EP practice more toward a clinical rather than an educational direction. This potential shift warrants further investigation in future research.

## Data Availability

The datasets presented in this article are not readily available because we have not obtained permission to share our data material. Requests to access the datasets should be directed to thoszu@ikp.aau.dk.
